# Adenosine A_2A_ Receptors as Biomarkers of Brain Diseases

**DOI:** 10.3389/fnins.2021.702581

**Published:** 2021-07-16

**Authors:** Ana Moreira-de-Sá, Vanessa S. Lourenço, Paula M. Canas, Rodrigo A. Cunha

**Affiliations:** ^1^CNC—Center for Neuroscience and Cell Biology, University of Coimbra, Coimbra, Portugal; ^2^Faculty of Medicine, University of Coimbra, Coimbra, Portugal

**Keywords:** adenosine A_2A_ receptors, central nervous system, antagonism, caffeine, biomarkers, polymorphisms

## Abstract

Extracellular adenosine is produced with increased metabolic activity or stress, acting as a paracrine signal of cellular effort. Adenosine receptors are most abundant in the brain, where adenosine acts through inhibitory A_1_ receptors to decrease activity/noise and through facilitatory A_2A_ receptors (A_2A_R) to promote plastic changes in physiological conditions. By bolstering glutamate excitotoxicity and neuroinflammation, A_2A_R also contribute to synaptic and neuronal damage, as heralded by the neuroprotection afforded by the genetic or pharmacological blockade of A_2A_R in animal models of ischemia, traumatic brain injury, convulsions/epilepsy, repeated stress or Alzheimer’s or Parkinson’s diseases. A_2A_R overfunction is not only necessary for the expression of brain damage but is actually sufficient to trigger brain dysfunction in the absence of brain insults or other disease triggers. Furthermore, A_2A_R overfunction seems to be an early event in the demise of brain diseases, which involves an increased formation of ATP-derived adenosine and an up-regulation of A_2A_R. This prompts the novel hypothesis that the evaluation of A_2A_R density in afflicted brain circuits may become an important biomarker of susceptibility and evolution of brain diseases once faithful PET ligands are optimized. Additional relevant biomarkers would be measuring the extracellular ATP and/or adenosine levels with selective dyes, to identify stressed regions in the brain. A_2A_R display several polymorphisms in humans and preliminary studies have associated different A_2A_R polymorphisms with altered morphofunctional brain endpoints associated with neuropsychiatric diseases. This further prompts the interest in exploiting A_2A_R polymorphic analysis as an ancillary biomarker of susceptibility/evolution of brain diseases.

## Introduction

The increased use of intracellular ATP, either because of increased workload or need to cope with stressful conditions, is a main source of increased extracellular levels of adenosine, which generally acts as a paracrine allostatic regulator by locally decreasing metabolism through inhibitory A_1_ receptors (A_1_R) and increasing metabolic supply through A_2A_R (Agostinho et al., 2020). Adenosine receptors are most abundant in the brain, where adenosine fulfills a role as neuromodulator apart from its general paracrine allostatic role: post-synaptic as well as astrocytic integrative activity are major contributors of an adenosine tone acting through inhibitory A_1_ receptors to decrease activity/noise in excitatory synapses; ATP release, characteristic of increased firing rate conditions associated with synaptic plasticity, is the major source of a second pool of synaptic extracellular adenosine selectively activating facilitatory A_2A_ receptors (A_2A_R) to promote synaptic plastic changes in physiological conditions (Cunha, 2016). However, ATP is also a general danger signal in the brain (Rodrigues et al., 2015), acting through a variety of ATP/ADP-activated P_2_ receptors to re-shape the function of astrocytes and microglia to cope with potential threats (Agostinho et al., 2020). Such threats also require adaptive plastic changes in neuronal circuits, which may explain the increased extracellular formation of ATP-derived adenosine by ecto-nucleotidases, with a burst of its rate-limiting step—ecto-5′-nucleotidase or CD73 (Cunha, 2001)—under noxious brain conditions to sustain an overfunction of A_2A_R that contributes to synaptotoxicity and neurotoxicity in different brain diseases (Cunha, 2016).

## Adenosine A_2A_ Receptors in Brain Diseases

Upon acute brain injury, probably best exemplified by an ischemic brain stroke, concurrent pharmacological and genetic evidence show that A_2A_R blockade affords a robust neuroprotection (reviewed in Chen and Pedata, 2008). In parallel, ischemia is accompanied by ATP release (Melani et al., 2005) and up-regulation of CD73 (Braun et al., 1997), thus increasing the formation of extracellular ATP-derived adenosine (Koos et al., 1997; Chu et al., 2014). Likewise, seizure-like activity characteristic of epileptic conditions triggers a neurodegeneration that is critically controlled by pharmacological or genetic A_2A_R blockade (Canas et al., 2018). Seizure activity also increases ATP release (Wieraszko and Seyfried, 1989) and up-regulates CD73 (e.g., Schoen et al., 1999; Rebola et al., 2003), increasing the contribution of extracellular ATP-derived adenosine formation to overactivate A_2A_R (reviewed in Tescarollo et al., 2020). A_2A_R blockade also attenuates brain damage following traumatic brain injury (TBI) (e.g., Li et al., 2009); TBI also bolsters the release of ATP (Faroqi et al., 2021) and CD73 levels (Zheng et al., 2020), although the contribution of extracellular ATP-derived adenosine has not yet been tested in TBI.

Overall, this evidence is compatible with an increase of extracellular adenosine, namely extracellular ATP-derived adenosine, leading to an overactivation of A_2A_R that contributes for brain dysfunction upon acute brain injury. A similar scenario seems to occur in chronic brain conditions. Thus, the pharmacological or genetic blockade of A_2A_R affords a consistent neuroprotection in animal models of Alzheimer’s disease (AD) (e.g., Canas et al., 2009; Laurent et al., 2016; Viana da Silva et al., 2016), Parkinson’s disease (PD) (reviewed in Schwarzschild et al., 2006)—where A_2A_R antagonists were approved by the US-FDA as novel anti-Parkinsonian drugs (Chen and Cunha, 2020), repeated stress/depression (Batalha et al., 2013; Kaster et al., 2015; Padilla et al., 2018), Machado-Joseph disease (Gonçalves et al., 2013), amyotrophic lateral sclerosis (ALS) (Ng et al., 2015; Rei et al., 2020; Seven et al., 2020), Angelman syndrome (Moreira-de-Sá et al., 2020, 2021), or glaucoma-like disorders (Madeira et al., 2015). Most of these chronic neuropsychiatric conditions are also associated with increased release of ATP, as occurs in animal models of AD (Gonçalves et al., 2019), PD (Carmo et al., 2019; Meng et al., 2019) or as concluded by the anti-depressant effects of P2 receptor antagonists (Ribeiro et al., 2019; but see Cao et al., 2013). Moreover, there is an increased contribution of extracellular ATP-derived adenosine for A_2A_R overactivation in chronic brain diseases, as best heralded by the observation that CD73 knockout mice phenocopy A_2A_R knockout mice (Augusto et al., 2013; Carmo et al., 2019; Gonçalves et al., 2019).

A_2A_R overactivation is not only necessary, but actually sufficient to trigger brain dysfunction, as concluded from the observation that the pharmacological overactivation of A_2A_R (Pagnussat et al., 2015), the optogenetic activation of A_2A_R transducing system (Li et al., 2015) or the over-expression of A_2A_R in the hippocampus (Coelho et al., 2014; Carvalho et al., 2019; Temido-Ferreira et al., 2020) are sufficient to trigger or aggravate brain dysfunction. Notably, A_2A_R overfunction seems to be an early event in different brain disorders (reviewed in Cunha, 2016), although A_2A_R antagonists seem to maintain their neuroprotective profile after the establishment of symptoms (e.g., Kaster et al., 2015; Faivre et al., 2018; Orr et al., 2018; Silva et al., 2018).

The tight association between increased release of ATP and its extracellular catabolism to overactivate A_2A_R as part of the expression of neuronal dysfunction at the onset and throughout the evolution of several brain diseases prompts exploiting this danger signaling pathway as new biomarkers to identify dysfunctional brain circuits in brain diseases. Although the tools are yet to developed, it may be promising to devise soluble sensors to detect altered levels of extracellular ATP to allow an *in vivo* estimate of brain circuits undergoing a particular purinergic pressure and, consequently, are at risk of undergoing dysfunction. An alternative could be the development of PET ligands (not yet available) to assess the density of CD73, which is paramount to link ATP upsurge with the selective overactivation of A_2A_R; CD73 seems to be consistently up-regulated upon brain stressful conditions and may be a selective biomarker of glia and synapses undergoing adaptive processes (Schoen and Kreutzberg, 1997).

## Up-Regulation of Adenosine A_2A_ Receptors in Brain Diseases

The A_2A_R overactivation associated with brain dysfunction and disease is not only sustained by an increased bioavailability of the trigger of A_2A_R—ATP-derived extracellular adenosine—but also involves an up-regulation of A_2A_R in the afflicted brain areas (reviewed in Cunha, 2016). Indeed, an increased density of cortical A_2A_R has been reported in animal models of epilepsy (Rebola et al., 2005; Cognato et al., 2010; Canas et al., 2018; Crespo et al., 2018), Rasmussen’s encephalopathy (He et al., 2020), TBI (Zhao et al., 2017), AD (Espinosa et al., 2013; Viana da Silva et al., 2016; Silva et al., 2018), Lyme neuroborreliosis (Smith et al., 2014), ALS (Seven et al., 2020), or chronic stress/depression (Kaster et al., 2015; Machado et al., 2017), as well as in the diseased human brain (Albasanz et al., 2008; Temido-Ferreira et al., 2020). Likewise, A_2A_R levels are also increased in the cerebellum of Machado-Joseph’s ataxic mice (Gonçalves et al., 2013) and in the amygdala or fear-conditioned mice (Simões et al., 2016). A_2A_R up-regulation is in fact an upsurge since it occurs shortly (within hours) after abnormal neuronal function (i.e., convulsions; Canas et al., 2018), but it gradually increases with aggravation of brain dysfunction (Temido-Ferreira et al., 2020). A_2A_R up-regulation mostly occurs in synapses, in accordance with the involvement of synaptic alterations at the onset of most brain diseases (e.g., Rebola et al., 2005; Kaster et al., 2015; Viana da Silva et al., 2016; Canas et al., 2018), but is also observed in glia cells in the progression of chronic brain diseases (Matos et al., 2012; Orr et al., 2015; Barros-Barbosa et al., 2016; Patodia et al., 2020). It is still unclear if this A_2A_R up-regulation only involves an increased readout of A_2A_R mRNAs (Canas et al., 2018) or also involves an overexpression of A_2A_R mRNA, which has been reported in the dysfunctional or diseased brain (e.g., Costenla et al., 2011; Espinosa et al., 2013; Hu et al., 2016; Dias et al., 2021). In fact, the triggers and mechanisms of this A_2A_R up-regulation in the diseased brain are essentially unknown. The A_2A_R gene in both rodents and humans has a complex promoter region and can give raise to multiple transcripts (Peterfreund et al., 1996; Lee et al., 2003a; Yu et al., 2004; Kreth et al., 2008; Huin et al., 2019). Although multiple controllers of the A_2A_R gene have been proposed, such as methylation patterns of the promoter (Falconi et al., 2019; Micioni Di Bonaventura et al., 2019), transcription factors ZBP-89 and Yin Yang-1 (Buira et al., 2010), microRNAs (e.g., Heyn et al., 2012; Villar-Menéndez et al., 2014; Zhao et al., 2015; Tian et al., 2016), NFk-B (Morello et al., 2006), cAMP-response element-binding protein (Chiang et al., 2005), hypoxia inducible factor-2α (Ahmad et al., 2009; Brown et al., 2011), AP1 transcription factor (Kobayashi and Millhorn, 1999; Lee et al., 2014), or nuclear factor 1 (Lee et al., 2003b), the regulation of the relative expression of these transcripts is largely unknown (Yu et al., 2004; Huin et al., 2019) and little is also known about the relative stability of the different mRNA transcripts. This is certainly an area of research that might open new avenues to design neuroprotective strategies linked to A_2A_R.

The association of A_2A_R up-regulation with brain diseases offers another promising opportunity to develop informative biomarkers of the susceptibility and/or evolution of different brain diseases once PET ligands are optimized to detect extra-striatal A_2A_R. In fact, A_2A_R throughout the brain are most abundant in the striatum (reviewed in Svenningsson et al., 1999) and the available PET ligands have been optimized to detect striatal A_2A_R (e.g., Mishina et al., 2011; Ishibashi et al., 2018); however, this population of A_2A_R has a different pharmacology (Orrú et al., 2011; Cunha, 2016), a different adaptive profile (Cunha et al., 1995) and a different role in most brain conditions (Shen et al., 2008, 2013; Yu et al., 2008; Wei et al., 2014). Thus, it is likely that the currently available PET ligands might not be useful to assess modifications of extra-striatal A_2A_R. New cortical A_2A_R-directed PET ligands need to be designed based on the particular properties and interacting partners of cortical A_2A_R (reviewed in Franco et al., 2020) to allow an *in vivo* detection of A_2A_R upsurge as potential general biomarkers of brain dysfunction (Sun et al., 2020).

A_2A_R are not only located in the brain, but are also present in several peripheral tissues, namely in different blood cells such as leukocytes and platelets (reviewed in Gessi et al., 2000). Based on the association of brain diseases with A_2A_R up-regulation in afflicted brain regions, several studies explored if A_2A_R in blood cells could be biomarkers of brain diseases, such as AD (Arosio et al., 2010, 2016; Merighi et al., 2021), PD (Falconi et al., 2019), or ALS (Vincenzi et al., 2013). However, only the understanding of the mechanisms underlying A_2A_R up-regulation in brain diseases will allow providing a rationale (or lack of thereof) to consider alterations of the density of peripheral A_2A_R as valid readouts of altered A_2A_R density that occurs selectively in afflicted brain circuits in the diseased brain.

## Polymorphisms of Adenosine A_2A_ Receptors and Brain Diseases

The gene encoding human A_2A_R (ADORA2A gene) harbors several single nucleotide polymorphisms (SPNs), which have been associated to an altered susceptibility to several neuropsychiatric and neurodegenerative disorders (Huin et al., 2019). In fact, as listed in Tables 1A,B, naturally occurring variabilities in the ADORA2A gene collectively influence predisposition risk and even age of onset for several CNS disorders as well as individual susceptibility to the anxiogenic and sleep-related consequences of caffeine. Although, it is still unknown if the different A_2A_R polymorphisms are associated with a different expression, subcellular location, trafficking, heteromerization or pharmacological properties of A_2A_R, the relation between A_2A_R polymorphisms and the susceptibility and age of onset of brain dysfunction prompts the interest in exploiting A_2A_R polymorphic analysis as an ancillary biomarker of susceptibility/evolution of brain diseases.

**TABLE 1:** Summary of the reported associations between known polymorphic variants of the human adenosine A_2A_ receptor gene (*ADORA2A*) and different response susceptibility to either pathological threats **(A)** or distinct physiological responses to external stimulus **(B)**.

(A) SNP	Type/Position	Risk allele/Genotype	Associated CNS Disorder	References
rs5751876	Exon 2	TT	Huntington’s disease (significant variability in age of onset)	Taherzadeh-Fard et al., 2010
		T	Susceptibility locus for Panic Disorder and Agoraphobia	Deckert et al., 1998; Hamilton et al., 2004; Domschke et al., 2012
		T	Prevalent in Gilles de la Tourette syndrome (GTS) patients	Janik et al., 2015
rs2298383	5′ UTR	TT	Huntington’s disease (significant variability in age of onset)	Taherzadeh-Fard et al., 2010
		CT	Higher predisposition for Childhood Epilepsy (CE)	Fan et al., 2020
		CC	Greater risk for CE patients to develop comorbid neurologic disorders	Fan et al., 2020
		CC/CT	Increased risk of Depression, Attention Deficits and Sleep-disturbances	Oliveira et al., 2019
		CC	Possible risk factor for Schizophrenia	Miao et al., 2019
rs2236624	Intron 4	CC	Associated with Autism Spectrum Disorders symptom severity	Freitag et al., 2010
rs71651683	5′ UTR	T	Inverse association with the likelihood to develop Parkinson’s disease (∼49%)	Popat et al., 2011
rs5996696	Promoter variant region	C	Inverse association with the likelihood to develop Parkinson’s disease (∼30%)	Popat et al., 2011
rs2298383, rs5751876, rs35320474, and rs4822492	5′ UTR, Exon 2, Exon 4, and 3′ UTR	C, T, deletion and C, respectively, (Haplotype A)	Predisposition of children to develop Acute Encephalopathy with biphasic seizures and late reduced diffusion (AESD)	Shinohara et al., 2013

**(B) SNP**	**Type/Position**	**Risk allele/Genotype**	**Physiological Response to Stimuli**	**References**

rs5751876	Exon 2	TT	Significant enhancement of caffeine-induced anxiety	Alsene et al., 2003; Childs et al., 2008
		TT	Associated with an overall lower caffeine intake and a prospective lesser vulnerability to caffeine dependence	Cornelis et al., 2007
		TT	Highest startle magnitudes upon caffeine administration in response to unpleasant pictures (maladaptive emotional processing)	Domschke et al., 2012
		TT	Associated with an ergogenic beneficial effect upon caffeine consumption	Loy et al., 2015
		TT	Higher anxiogenic response susceptibility to caffeine ingestion in usual non-consumers or low consumers (<40 mg per day), but no significant correlation with habitual caffeine intake	Rogers et al., 2010
		C	Sleep disturbances (and insomnia) triggered by caffeine intake, higher β-activity in non-REM sleep	Rétey et al., 2007
		T	Lower total sleep time in habitual low caffeine consumers	Erblang et al., 2019
		T	Increased sleep latency associated with caffeine consumption, lower percentage of N3 sleep stage	Nunes et al., 2017
rs2298383	5′ UTR	CC	Significant enhancement of caffeine-induced anxiety	Childs et al., 2008
		C	Lower total sleep time in habitual low caffeine consumers	Erblang et al., 2019
rs4822492	3′ UTR	CC	Significant enhancement of caffeine-induced anxiety	Childs et al., 2008
		CC	Lower total sleep time in habitual low caffeine consumers	Erblang et al., 2019
rs3761422	5′ UTR	TT	Greater increase in anxiety upon caffeine ingestion in habitual non-consumers or low consumers (<40 mg per day)	Rogers et al., 2010
		T	Lower total sleep time in habitual low caffeine consumers	Erblang et al., 2019

## Discussion

A_2A_R overfunction is necessary and actually sufficient for the expression of neuronal dysfunction upon brain diseases. In particular, A_2A_R overfunction associated with aberrant synaptic plasticity and synaptotoxicity seems to be associated with the onset of symptoms of brain diseases. However, some of these symptoms are comorbidities of other brain diseases, associated with their aggravation, which often involves a spreading of neuroinflammation, also known to be controlled by A_2A_R. Thus, it is also likely that A_2A_R overfunction might be also associated with the evolution of brain diseases. These neuropathological roles of A_2A_R prompts considering the exploitation of this system as candidate biomarkers of the susceptibility and evolution of brain diseases. The development of PET ligand with adequate signal-to-noise ratio and selectivity to detect the relevant extra-striatal A_2A_R may allow a minimally invasive assessment of A_2A_R in different brain regions. This may be complemented by the definition of A_2A_R polymorphisms as an ancillary biomarker for the susceptibility and evolution of brain diseases, which still requires a firm establishment of structural-functional relationships between A_2A_R polymorphisms and brain dysfunction. Finally, the future development of PET-based sensors of extracellular ATP and/or adenosine may well be of additional interest as a biomarker of the status of brain diseases to be used in complement of other available methods.

## Author Contributions

All authors contribute to the organization and writing of the review.

## Conflict of Interest

RC is a scientific consultant for the Institute for Scientific Information on Coffee. The remaining authors declare that the research was conducted in the absence of any commercial or financial relationships that could be construed as a potential conflict of interest.
